# Structure of Utp21 Tandem WD Domain Provides Insight into the Organization of the UTPB Complex Involved in Ribosome Synthesis

**DOI:** 10.1371/journal.pone.0086540

**Published:** 2014-01-21

**Authors:** Cheng Zhang, Jinzhong Lin, Weixiao Liu, Xining Chen, Rongchang Chen, Keqiong Ye

**Affiliations:** 1 Institute of Developmental Biology and Molecular Medicine, School of Life Science, Fudan University, Shanghai, China; 2 National Institute of Biological Sciences, Beijing, Beijing, China; Univ. of Edinburgh, United Kingdom

## Abstract

Assembly of the eukaryotic ribosome requires a large number of *trans*-acting proteins and small nucleolar RNAs that transiently associate with the precursor rRNA to facilitate its modification, processing and binding with ribosomal proteins. UTPB is a large evolutionarily conserved complex in the 90S small subunit processome that mediates early processing of 18S rRNA. UTPB consists of six proteins Utp1/Pwp1, Utp6, Utp12/Dip2, Utp13, Utp18 and Utp21 and has abundant WD domains. Here, we determined the crystal structure of the tandem WD domain of yeast Utp21 at 2.1 Å resolution, revealing two open-clamshell-shaped β-propellers. The bottom faces of both WD domains harbor several conserved patches that potentially function as molecular binding sites. We show that residues 100–190 of Utp18 bind to the tandem WD domain of Utp21. Structural mapping of previous crosslinking data shows that the WD domains of Utp18 and Utp1 are organized on two opposite sides of the Utp21 WD domains. This study reports the first structure of a UTPB component and provides insight into the structural organization of the UTPB complex.

## Introduction

Ribosomes are large molecular machines that consist of four rRNAs and ∼80 ribosomal proteins in eukaryotes. These components are assembled in a highly complicated and dynamic process [1], [2]. Ribosome synthesis starts with RNA polymerase I-mediated transcription of a long precursor rRNA (pre-rRNA) in the nucleolus. The pre-rRNA is then extensively modified and processed to remove spacer sequences, producing the 18S rRNA in the small ribosomal subunit, and the 5.8S and 25S rRNAs in the large ribosomal subunit. The 5S rRNA in the large subunit is transcribed by RNA polymerase III from separate genes.

Genetic and biochemical studies have found over two hundred proteins involved in ribosome synthesis in *Saccharomyces cerevisiae*. Most of them are conserved throughout eukaryotes, although humans have additional ribosome synthesis factors that are absent in yeast [3], [4]. Some of these factors are enzymes, such as helicases, ATPases, GTPases, kinases, nucleases and RNA modification enzymes, but the majority appear to be scaffolding proteins that contain protein- or RNA-binding domains or degenerated enzyme domains [5], [6]. These assembly factors transiently associate with the pre-rRNA to form a series of pre-ribosomal particles, mediating modification and processing of the pre-rRNA and coordinating rRNA folding and ribosomal protein binding. During transcription of pre-rRNA, the formation of a large knob can be observed by electron microscopy at the 5′ pre-18S portion of the nascent pre-rRNAs [7]. This particle, known as the 90S pre-ribosome or the small subunit (SSU) processome, is responsible for early processing of the 18S rRNA at sites A0, A1 and A2 [8], [9]. The A0 and A1 cleavages in the 5′ external transcribed spacer (5′-ETS) generate the 5′-end of 18S, and the A2 cleavage in the internal transcribed spacer 1 (ITS-1) separates two pre-rRNA intermediates destined for the small and large subunits.

The SSU processome is an enormous particle that contains 35S pre-rRNA, U3 snoRNA, ∼50 assembly factors and a subgroup of r-proteins [2], [8], [9]. The structure and assembly pathway of the SSU processome are currently poorly understood. One important step in dissecting the structure of the SSU processome is to characterize the structure of its individual components and subcomplexes and to identify the interaction network among its components. A few subcomplexes including U3 snoRNP, UTPA, UTPB and UTPC have been biochemically purified independently from the SSU processome [8], [10], [11]. These subcomplexes appear to function as individual modules and assemble into pre-ribosomes in a hierarchical order [12], [13].

UTPB is a large complex composed of six proteins Utp1/Pwp2, Utp6, Utp12/Dip2, Utp13, Utp18 and Utp21 [8], [10]. It has a total molecular weight of 525 kDa, assuming there is a single copy of each protein. All these proteins are essential for yeast viability and are highly conserved from yeast to humans, underscoring their functional importance. Mutations in WDR36, the human UTP21 gene, have been associated with primary open angle glaucoma, a leading cause of blindness [14]. Haploinsufficiency of UTP6 may contribute to the severity of neurofibromatosis type 1 [15].

The WD domain (aka WD40 domain) is composed of 40–60 residue long WD repeats arranged in a β-propeller structure and commonly mediates protein-protein interactions in diverse cellular processes [16]. The whole UTPB complex possesses a remarkable number of WD domains, namely, nine in five proteins. Utp1, Utp12, Utp13 and Utp21 all contain two WD domains (WD1 and WD2) followed by 170–260 C-terminal residues that contain no recognizable domain (hereafter called the C-terminal domain, CTD). Utp18 has a single WD domain preceded by an N-terminal region. Utp6, the only component without a WD domain, possesses a HAT domain, which is also a protein-interaction domain. The abundant WD domains in UTPB may mediate interactions within the complex and with other assembly factors.

Interactions between the six UTPB proteins have been mapped by yeast two-hybrid assay, but the precise domain of each protein participating in binding is mostly unclear [17]. More recently, chemical crosslinking and mass spectrometry analysis of a recombinant UTPB complex identified 71 high-quality crosslinks between lysine pairs, including 50 intramolecular and 21 intermolecular crosslinks [18]. These crosslinks provide valuable structural information regarding UTPB but are of limited use due to the current lack of a high-resolution structure for any component of UTPB.

As part of an effort to understand the structure of UTPB, we have determined the crystal structure of the tandem WD domain of Utp21, the first structure of a UTPB component. We further identified a region of Utp18 that mediates the interaction with Utp21. The structure allows us to integrate available crosslinking and interaction data to obtain a better understanding of the organization of UTPB.

## Results and Discussion

### Interaction between Utp18 and Utp21

**Figure 1:**
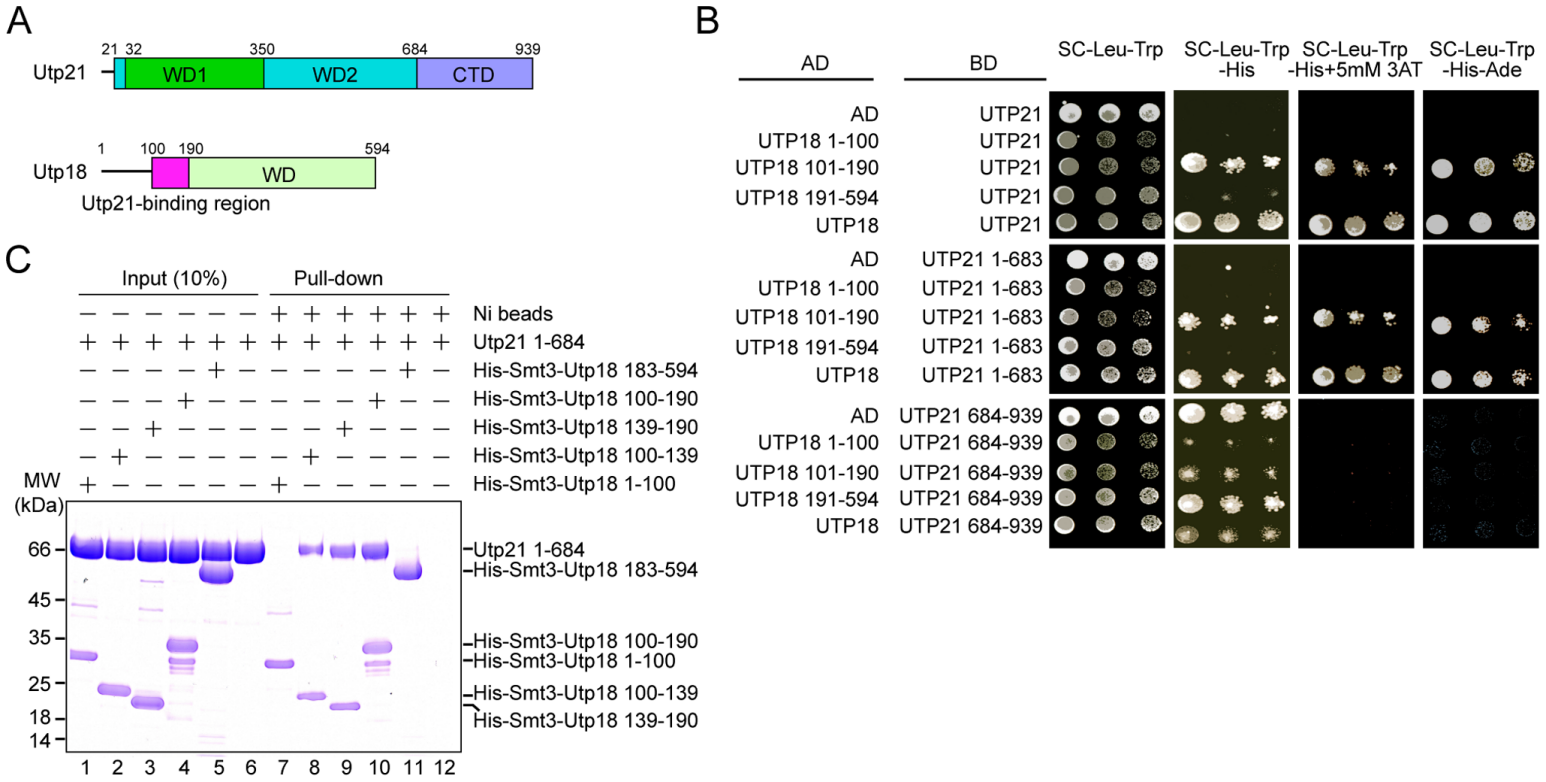
Interaction between Utp21 and Utp18. (A) Domain diagrams of Utp21 and Utp18. The WD1 and WD2 domains, which were resolved in the crystal structure, and the C-terminal domain (CTD) are labeled in Utp21. The predicted WD domain and the Utp21-binding motif are labeled in Utp18. (B) Yeast two-hybrid assay. The indicated genes were fused to the activation domain (AD) and GAL4 DNA-binding domain (BD) and co-expressed in AH109 cells. Ten-fold serial dilutions of yeast cells were spotted onto plates containing the indicated SC dropout media and grown at 30°C for 3 d. (C) Ni-bead pull-down assay. Utp21 1–684 (1.9 nmol) and the indicated His-Smt3-tagged Utp18 fragments (0.95 nmol) were mixed and incubated with Ni Sepharose beads. The input (10%) and bound proteins were analyzed by SDS-PAGE with Coomassie staining. The positions of molecular weight markers are indicated on the left. Note that all Utp18 fragments containing residues 100–139 migrate more slowly than expected.

**Figure 2:**
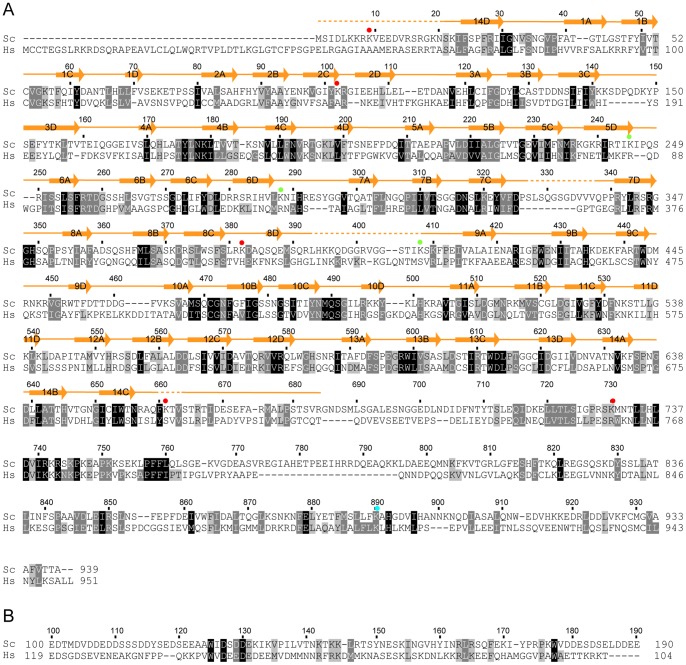
Multiple sequence alignments of Utp21 and Utp18. The alignments were conducted for 187 Utp21 sequences (A) and 193 Utp18 sequences (B). Only the sequences of *S. cerevisiae* (Sc) and *H. sapiens* (Hs) are displayed. Residues that are conserved in at least 95%, 80% and 70% of all the aligned sequences are shaded in black, gray and light gray, respectively. Secondary structural elements observed in the crystal structure of Utp21 are shown on the top of the alignment. Dots denote disordered regions. The Utp21 residues that are crosslinked to Utp1 (red), Utp18 (green) and Utp12 (cyan) are labeled with circles.

Utp21 has been shown to interact with Utp18 in a two-hybrid assay [17]. To locate which portion of each protein interacts with one another, Utp21 and Utp18 were divided into smaller fragments according to sequence-based domain prediction (Figure 1A and 2) and analyzed by two-hybrid assays under different stringency conditions (Figure 1B). The results verify that Utp18 interacts strongly with Utp21. Among the three fragments of Utp18 tested, the fragment with residues 101–190 bound Utp21 similarly to full-length Utp18, whereas no interaction was found for residues 1–100 and the isolated WD domain (residues 191–594) of Utp18. Analysis of two Utp21 fragments shows that the tandem WD domain (residues 1–683), but not the CTD (residues 684–939), interacted with Utp18. The CTD of Utp21 displayed some auto-activation under the low stringent growth condition.

To confirm the physical interaction between Utp21 and Utp18, we conducted pull-down experiments using recombinant proteins (Figure 1C). The tandem WD domain of Utp21 was expressed as a GST fusion and the GST-tag was cleaved during purification. The Utp18 fragments were expressed with an N-terminal His_6_-tag. The full-length Utp21, the full-length Utp18 and the CTD of Utp21 could not be examined due to protein expression problems. The Utp21 and Utp18 proteins in different combinations were mixed and pulled down using Ni beads. SDS-PAGE analyses show that Utp18 100–190, but not Utp18 1–100 and Utp18 183–594, efficiently copurified with Utp21 1–684 (Figure 1C, lanes 1,4,5), in agreement with the two-hybrid results. The detected interaction was specific because Utp21 1–684 alone did not bind to the Ni beads (Figure 1C, lane 6). To narrow down the interacting region of Utp18, two shorter fragments consisting of residues 100–139 and 139–190 were assessed. Both fragments bound Utp21 1–684 yet appeared to bind more weakly than the longer Utp18 100–190. The two-hybrid and pull-down results demonstrate that residues 100–190 of Utp18 interact with the tandem WD domain of Utp21. A few conserved residues are distributed in the Utp21-binding region of Utp18, supporting its functional role (Figure 2B).

### Structural Determination of the Utp21 Tandem WD Domain

**Figure 3:**
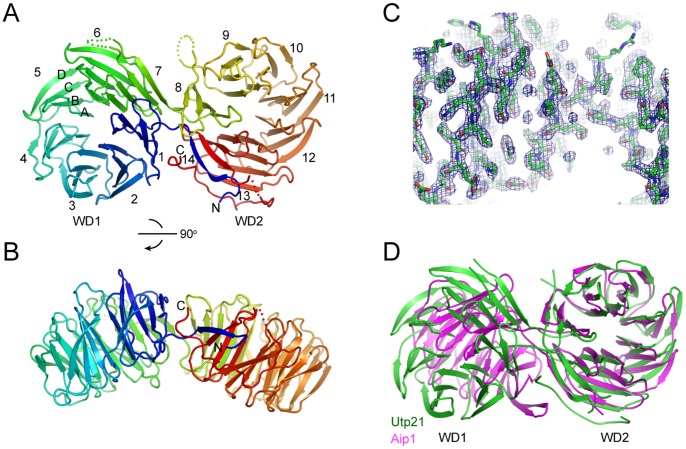
Structure of Utp21 tandem WD domains. (A) Ribbon representation of the Utp21 structure. The 14 numbered blades, four β-strands in blade 5 and the N- and C-termini are labeled. The protein chain is colored blue to red from the N- to C-termini. Dots denote disordered regions. (B) A view rotated by 90°. (C) The 2*f*_o_-*f*_c_ electron density map contoured at the 1.5 σ level. (D) Structural superposition of the WD2 domains of Utp21 and Aip1. Note the different orientations of the WD1 domains following the alignment.

We co-expressed and co-purified Utp21 1–684 with each of the three Utp18 fragments (100–190, 100–139 and 139–190) and screened these complexes for crystal formation. The complex that copurified with Utp18 100–139 was the only one that crystallized. The structure was determined by single isomorphous replacement with anomalous signal (SIRAS) phasing using a native and Se-derivative dataset. The current model was refined to 2.1 Å resolution with *R*_work_ and *R*_free_ values of 0.187 and 0.224, respectively (Figure 3A–C, Table 1). The crystal contains one molecule of Utp21, but no Utp18 molecule, in the asymmetric unit. Residues 1–19, 327–337, 393–408 and 660–663 of Utp21 were not modeled due to missing electron density. The Utp18 fragment apparently dissociated or degraded during crystallization. The other two Utp18 fragments bound Utp21 more tightly than the 100–139 fragment but unfortunately could not be crystallized.

**Table 1:** Data collection and refinement statistics.

Crystal form	Se-labeled	Native
Data collection		
Space group	P1	P1
Cell dimensions		
a, b, c (Å)	35.7, 64.3, 82.4	35.5, 63.5, 82.5
α, β, γ (°)	111.5, 95.5, 98.3	111.0, 95.8, 97.9
Wavelength (Å)	0.97934	0.97915
Resolution range (Å)	50–2.5(2.54–2.5)	50–2.1(2.14–2.1)
Unique reflections	22617(1088)	37483(1852)
Redundancy	3.6(3.4)	2.6(2.4)
<*I*>/<σ(*I*)>	18.8(3.7)	13.7(2.8)
Completeness (%)	97.0(91.4)	97.9(97.3)
*R*_merge_	0.117(0.485)	0.142(0.478)
Structure refinement		
Resolution range (Å)		20–2.1(2.16–2.1)
No. reflections		35416
No. atoms		5381
Protein		4974
Solvent		394
Citrate		13
*R*_work_		0.187(0.223)
*R*_free_		0.224(0.294)
Mean B factor (Å^2^)		25.8
Protein		25.4
Solvent		29.9
Citrate		41.3
Rmsd bond length (Å)		0.003
Rmsd bond angle (°)		0.834

Values in parentheses are for the data in the highest-resolution shell.

### Structural Description

The tandem WD domains of Utp21 fold into two 7-blade β-propellers, resembling an open clamshell (Figure 3A–B). Each blade is composed of four anti-parallel β-strands named A, B, C and D in the outward direction. Blades 1–7 form the N-terminal WD1 domain, and blades 8–14 form the C-terminal WD2 domain. The two WD domains are connected by the 14D-1A and 7D-8A loops. Individual WD repeats make up strand D of one blade and strands A, B and C of the successive blade (Figure 2A). Notably, strand D of the first repeat pairs with strand C of the last repeat, forming the so called “velcro” closure. Such a structural organization is conserved for single, double and triple WD domains [19], [20].

Only a few structures have been determined for tandem WD domain proteins, including Aip1 [20], entry 2YMU in the Protein Data Bank (Chaudhuri I. and Zeth K., unpublished) and the apoptotic protein Apaf1 [21]. Utp21 shares a similar overall topology with Aip1 and 2YMU but not with Apaf1. The relative orientation of the two β-propellers are variable among different structures. The two β-propellers of Utp21 are more open with an angle of ∼150° compared to those in Aip1 that have an angle of ∼110° (Figure 3D). In addition to the core β-propeller domains, Utp21 possesses an extra loop (residues 657–684) following the last strand, 14C. The loop wraps around the side of blades 13 and 14 and inserts into the inter-domain space, making contacts with blade 1.

### The Bottom Surfaces of each WD Domain Harbor Multiple Conserved Patches

**Figure 4:**
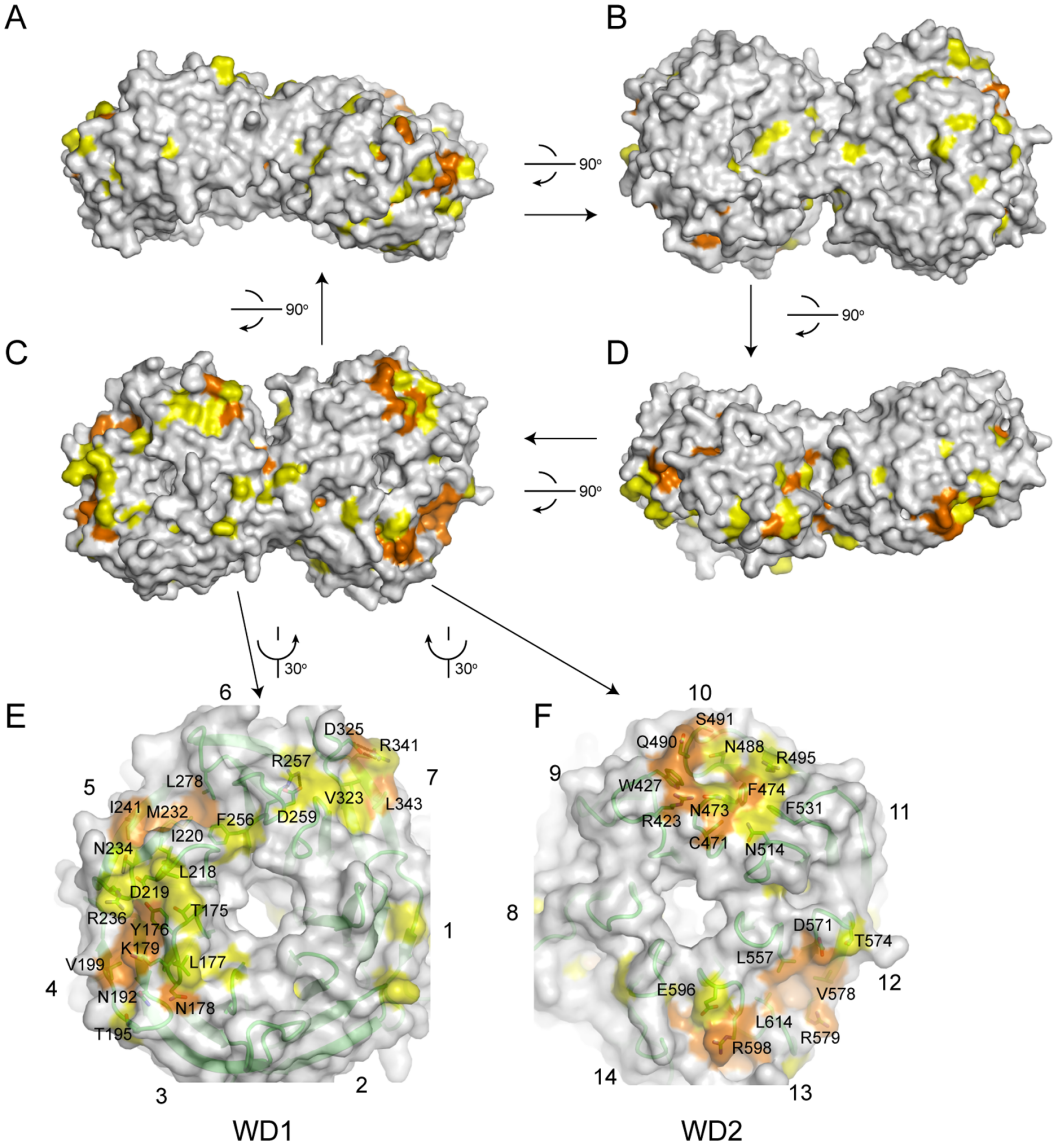
Conservation surface of Utp21 WD domains. (A–D) Surface representation of Utp21 WD domains. Residues with >95% and 95–80% conservation, as defined in Figure 2A, are colored in orange and yellow, respectively. The structure is consecutively rotated 90° along a horizontal axis in the paper plane to yield the four views. The views in C and A have the same orientations as those in Figure 3A and 3B, respectively. (E–F) Close-up views of conserved surface patches in the WD1 (E) and WD2 (F) domains. The conserved residues are shown as sticks and labeled. For a better view, the structure is rotated 30° counterclockwise (E) or clockwise (F) along a vertical axis in the paper.

Utp21 has been shown to interact with multiple proteins, including Utp18, Utp1, Utp12 and Utp6 in the UTPB complex and SSU biogenesis factors Sas10/Utp3 and Utp25 [17], [22]. These protein-binding sites are most likely conserved on the surface of the Utp21 structure. To gain insight into the potential protein-binding sites on the Utp21 WD domains, we mapped the residues that are conserved in at least 80% of 187 homologous Utp21 proteins from diverse eukaryotes onto the structure (Figure 4A–D).

Traditionally, the surface of WD propellers composed by the B-C and D-A loops is termed the top surface, whereas the opposite surface covered by the A-B and C-D loops is termed the bottom surface. The bottom surface of the WD1 domain displays an extensive conserved region that spans blades 4–7 (Figure 4E). The A-B and C-D loops of blades 4 and 5 contain dense conserved amino acids of mixed nature. The space between blades 5 and 6 creates a conserved hydrophobic groove. In addition, two salt bridges, R257-D259 and R341-D325, in blades 6 and 7 likely play a structural role.

The WD2 domain contains two separated conserved patches also on the bottom surface (Figure 4F). One is formed on blades 9–11 with concentrated hydrophobic, charged and polar residues. The other is located on blades 12 and 13 and includes a hydrophobic pocket surrounded by charged and polar residues.

In contrast to the conserved bottom surfaces, other surface areas are variable in either of the WD domains (Figure 4A–D). This suggests that the bottom surfaces of the two Utp21 WD domains are used for assembling multiple protein partners. The top surface is the most frequent protein-binding site in WD β-propellers, although all faces can be used for protein interactions [16].

### Insight into the Architecture of UTPB

**Figure 5:**
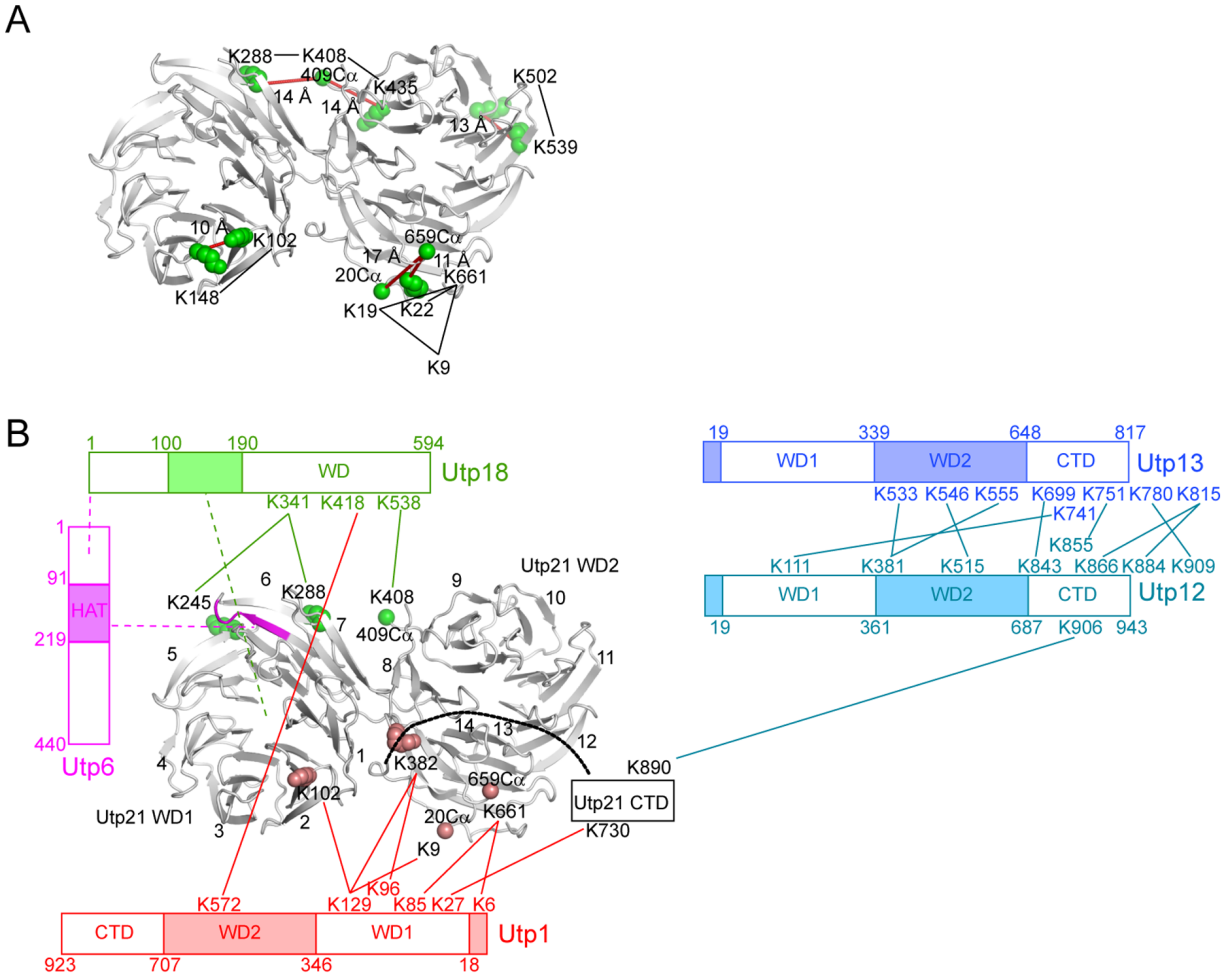
Organization of UTPB. (A) Mapping of intramolecular crosslinking sites on the Utp21 tandem WD domain structure. Crosslinked lysines are shown as green spheres. When a crosslinked lysine (K9, K19, K408 and K661) is disordered in the crystal structure, the Cα atom of its closest terminal residue (residues 20, 409 and 659) is displayed as a sphere as a reference point. Crosslinked lysines are connected by black lines. The Cα-Cα distances between crosslinked lysines or reference points are indicated and marked as red lines. (B) Architecture of the UTPB complex. The intermolecular crosslinks are mapped to the Utp21 tandem WD domain structure or shown on the domain diagrams of other proteins. Lines denote crosslinks. Dashed lines denote interactions demonstrated by yeast 2-hybrid or pull-down assays. Residues crosslinked to Utp18 are colored in green, residues crosslinked to Utp1 are colored in red, and residues 274–279, the key binding site of the Utp6 HAT domain, are colored in magenta.

The interaction and spatial proximity of the six UTPB proteins have been mapped to some extent [17], [18], [23]. The structure of the Utp21 WD domains now provides an opportunity to model the interaction data in a 3-dimensional framework. We previously crosslinked a purified recombinant UTPB complex with the amine-specific bivalent crosslinker BS3 and identified crosslinked peptides by mass spectrometry [18]. Among the 14 intramolecular crosslinks within Utp21, eight are derived from its WD domains. Structural mapping of these crosslinks reveals that all Cα-Cα distances between crosslinked residues are less than the theoretical upper limit of 24 Å (Figure 5A), indicating that the crosslinking data report authentic structural information.

In addition, Utp21 was found to form eleven intermolecular crosslinks with Utp18, Utp1 and Utp12 [18]. These crosslinks provide important distance constraints to derive the structural organization of UTPB (Figure 5B). The Utp18 residues K341 and K538 in the WD domain were found to crosslink to Utp21 residues K245, K288 and K408. The three crosslinked Utp21 residues are all located in the D-A loops of blades 5, 6 and 8 along the side surface of two WD domains. Our two-hybrid and pull-down results show that residues 100–190 of Utp18, but not its WD domain, have detectable interactions with the tandem WD domain of Utp21 (Figure 1). Therefore, the crosslinks between the Utp18 WD domain and the Utp21 WD domains should result from their spatial proximity, rather than from a stable interaction.

Utp6 has been shown to bind Utp18 via its N-terminal region and to bind Utp21 via its HAT domain [17]. Utp21 residues 274–279 constitute a key binding site for Utp6 [17]. The Utp21 structure reveals that residues 274–279 make up the 6C strand and the 6CD loop and are in close proximity to the Utp18 crosslinking sites. These interaction data suggest that Utp18 and Utp6 are neighbors and situated around blades 5, 6 and 8 of Utp21.

Four Utp1 residues (K6, K85, K96 and K129) mainly from the WD1 domain were previously shown to make six crosslinks with four residues (K9, K102, K382 and K661) in the WD domains of Utp21 (Figure 5B). In the Utp21 structure, K102 is located in the 2CD loop and K382 is situated in the 8CD loop. K9 and K661 are disordered, and their positions can be approximated by the closest terminal residues 20 and 659, respectively. These Utp1 crosslinking sites are clustered along the side surface of blades 2, 8 and 14, suggesting that Utp1, or more precisely its WD1 domain, is bound there. In addition, Utp1 K27 crosslinked with Utp21 K730 in the CTD. The CTD of Utp21, which lacks any structural information so far, is connected to the C-terminus of the WD2 domain, and would also be close to the Utp1 WD1 domain. In summary, structural mapping of the crosslinking data places the WD domains of Utp1 and Utp18 on two opposite sides of the Utp21 tandem WD domains.

## Conclusions

Understanding the structure of the entire UTPB complex is going to be a challenging task. A practical way to do so is to determine the structure of the individual domains and subcomplexes and to then assemble them with low-resolution constraints, such as crosslinking data, obtained from the entire complex. Toward this goal, we have analyzed the structure and interactions of the Utp21 tandem WD domains. Utp21 occupies a central position in the UTPB complex as it interacts with all of the other proteins except Utp13 [17].

The crystal structure of the Utp21 tandem WD domain reveals an overall topology similar to those found in Aip1 and 2YMU, indicating that this topology is the prevalent, although not exclusive (Apaf1 is an exception), way to arrange tandem WD domains. In UTPB, Utp1, Utp12 and Utp13 are predicted to also contain tandem WD domains, which the Utp21 structure could provide a good template to model. We have found several highly conserved patches on the bottom face of each of the Utp21 WD domains that are potential binding sites for other molecules. We identified residues 100–190 of Utp18 as interacting with the tandem WD domain of Utp21, but the structural details of the interaction await further investigation. Finally, the atomic structure of the Utp21 WD domains serves as a 3-dimensional model to interpret the previous crosslinking data. By determining additional structures of the individual components and subcomplexes of UTPB, we hope to assemble a complete UTPB structure in the future.

## Materials and Methods

### DNA Cloning and Protein Purification

The Utp21 and Utp18 genes were PCR-amplified from yeast genomic DNA. Gene cloning was conducted with the ligation-based or In-fusion (TaKaRa) method. The DNA of Utp21 1–684 was cloned into pGEX-6P-1 and expressed as a GST-fusion protein. The DNA of various Utp18 fragments was cloned into pET28a and expressed with an N-terminal His_6_-Smt3-tag.

To purify the Utp21/Utp18 complex, two plasmids encoding GST-Utp21 1–684 and one His_6_-Smt3-Utp18 fragment were co-transformed into *E. coli* strain BL21(DE3). The cells were cultured in LB medium, and protein expression was induced with 0.2 mM isopropyl β-D-1-thiogalactopyranoside for 12 h at 22°C. The cells were collected by centrifugation and resuspended in lysis buffer containing 25 mM Tris-HCl pH 7.5 and 500 mM NaCl. The cells were broken using a high-pressure JN-3000 cell disruptor (JNBIO) and clarified by centrifugation at 4°C. After incubation with Ulp1 for 4 h at 4°C to cleave the His_6_-Smt3 tag from Utp18, the supernatant was loaded onto a Glutathione Sepharose column followed by a wash with lysis buffer. The GST-fusion protein was incubated on-bead with PreScission protease for 8 h at 4°C. The released protein was eluted with lysis buffer, concentrated and further purified in a HiLoad 16/60 Superdex 200 PG column with buffer (10 mM Tris-HCl pH 7.5 and 200 mM NaCl). The pooled fractions of the target protein were concentrated to 5–10 mg/ml and stored at −80°C.

The selenomethionine(SeMet)-labeled protein was expressed in M9 minimal medium with inhibition of the methionine biosynthesis pathway [24] and purified in the same way as the native protein except that all buffers were supplemented with 0.5 mM Tris (2-carboxyethyl) phosphine hydrochloride.

### Crystallization and Structural Determination

The copurified Utp21 1–684/Utp18 100–139 complex was crystallized using the vapor diffusion sitting drop method at 20°C. One microliter of the protein sample (5.2 mg/ml in 10 mM Tris-HCl pH 7.5, 200 mM NaCl) was mixed with an equal volume of mother solution (0.15 M sodium citrate pH 8.5 and 15% PEG 3350). SeMet-labeled crystals were crystallized under the same conditions using native microcrystals as seed. The crystal was cryoprotected in 25% glycerol made from the mother solution.

All diffraction data were collected at the BL17U beamline of the Shanghai Synchrotron Radiation Facility (SSRF) and processed with HKL2000 [25]. One dataset was collected for the SeMet-labeled crystal to 2.5 Å resolution at a wavelength of 0.9794 Å. Another dataset was collected for the native isomorphous crystal to 2.1 Å resolution. The crystal belonged to space group P1 with one Utp21 molecule in the asymmetric unit (Table 1). The phases of structural factors were determined with SIRAS in SHARP [26], which employs SHELX/D for heavy atom search [27], and were improved by solvent modification. The model was built in COOT [28] and refined against a 2.1 Å resolution native dataset with refmac and Phenix [29], [30]. The final model contains Utp21 residues 20–326, 338–392, 409–659 and 664–684; 394 water molecules; and one citrate molecule. Analysis by RAMPAGE shows that 97.4% of the residues are in favored regions and that 2.6% are in allowed regions.

### Yeast Two-hybrid Assay

The yeast two-hybrid assay was performed using the MATCHMAKER GAL4 two-hybrid system (Clontech) as described [5]. Yeast AH109 cells were co-transformed with two plasmids containing genes fused to AD and BD, and selected in Synthetic Complete (SC) medium lacking Leu and Trp. Interactions between the bait and prey proteins were assayed in SC-Leu-Trp-His (low stringency) and SC-Leu-Trp-His-Ade (high stringency) media. In addition, 3-amino-1,2,4-triazole (3-AT) was added at 5 mM to the SC-Leu-Trp-His medium to increase selection stringency.

### Pull-down Assay

The Utp21 1–684 protein was purified as described above. The His_6_-Smt3-tagged Utp18 proteins were purified by HisTrap and Q chromatography. Concentrations of each protein were determined using the measured absorbance at 280 nm and the theoretic molar extinction coefficient. The Utp18 proteins (0.95 nmol) were incubated with Utp21 1–684 (1.9 nmol) for 2 h at 4°C and then bound to Ni Sepharose Fast Flow beads (GE Healthcare). The beads were washed with buffer (25 mM Tris-HCl pH 7.6, 500 mM NaCl and 10 mM imidazole) three times, followed by boiling in SDS loading buffer at 95°C for 5 min. The proteins were separated on an SDS-PAGE gel and stained with Coomassie blue.

### Accession Number

The coordinates and structural factors have been deposited into Protein Data Bank with the accession code 4NSX.

## References

[1] Henras AK, Soudet J, Gerus M, Lebaron S, Caizergues-Ferrer M, et al (2008) The post-transcriptional steps of eukaryotic ribosome biogenesis. Cell Mol Life Sci 65: 2334–2359. 18408888 10.1007/s00018-008-8027-0 PMC11131730

[2] Phipps KR, Charette JM, Baserga SJ (2011) The SSU processome in ribosome biogenesis - progress and prospects. Wiley Interdiscip Rev RNA 2: 1–21. 21318072 10.1002/wrna.57 PMC3035417

[3] Tafforeau L, Zorbas C, Langhendries JL, Mullineux ST, Stamatopoulou V, et al (2013) The complexity of human ribosome biogenesis revealed by systematic nucleolar screening of Pre-rRNA processing factors. Mol Cell 51: 539–551. 23973377 10.1016/j.molcel.2013.08.011

[4] Wild T, Horvath P, Wyler E, Widmann B, Badertscher L, et al (2010) A protein inventory of human ribosome biogenesis reveals an essential function of exportin 5 in 60S subunit export. PLoS Biol 8: e1000522. 21048991 10.1371/journal.pbio.1000522 PMC2964341

[5] Lin J, Lu J, Feng Y, Sun M, Ye K (2013) An RNA-binding complex involved in ribosome biogenesis contains a protein with homology to tRNA CCA-adding enzyme. PLoS Biol 11: e1001669. 24130456 10.1371/journal.pbio.1001669 PMC3794860

[6] Lu J, Sun M, Ye K (2013) Structural and functional analysis of Utp23, a yeast ribosome synthesis factor with degenerate PIN domain. RNA 19: 1815–1824. 24152547 10.1261/rna.040808.113 PMC3860261

[7] Osheim YN, French SL, Keck KM, Champion EA, Spasov K, et al (2004) Pre-18S ribosomal RNA is structurally compacted into the SSU processome prior to being cleaved from nascent transcripts in Saccharomyces cerevisiae. Mol Cell 16: 943–954. 15610737 10.1016/j.molcel.2004.11.031

[8] Grandi P, Rybin V, Bassler J, Petfalski E, Strauss D, et al (2002) 90S pre-ribosomes include the 35S pre-rRNA, the U3 snoRNP, and 40S subunit processing factors but predominantly lack 60S synthesis factors. Mol Cell 10: 105–115. 12150911 10.1016/s1097-2765(02)00579-8

[9] Dragon F, Gallagher JE, Compagnone-Post PA, Mitchell BM, Porwancher KA, et al (2002) A large nucleolar U3 ribonucleoprotein required for 18S ribosomal RNA biogenesis. Nature 417: 967–970. 12068309 10.1038/nature00769 PMC11487672

[10] Krogan NJ, Peng WT, Cagney G, Robinson MD, Haw R, et al (2004) High-definition macromolecular composition of yeast RNA-processing complexes. Mol Cell 13: 225–239. 14759368 10.1016/s1097-2765(04)00003-6

[11] Watkins NJ, Segault V, Charpentier B, Nottrott S, Fabrizio P, et al (2000) A common core RNP structure shared between the small nucleoar box C/D RNPs and the spliceosomal U4 snRNP. Cell 103: 457–466. 11081632 10.1016/s0092-8674(00)00137-9

[12] Perez-Fernandez J, Martin-Marcos P, Dosil M (2011) Elucidation of the assembly events required for the recruitment of Utp20, Imp4 and Bms1 onto nascent pre-ribosomes. Nucleic Acids Res 39: 8105–8121. 21724601 10.1093/nar/gkr508 PMC3185420

[13] Perez-Fernandez J, Roman A, De Las Rivas J, Bustelo XR, Dosil M (2007) The 90S preribosome is a multimodular structure that is assembled through a hierarchical mechanism. Mol Cell Biol 27: 5414–5429. 17515605 10.1128/MCB.00380-07 PMC1952102

[14] Monemi S, Spaeth G, DaSilva A, Popinchalk S, Ilitchev E, et al (2005) Identification of a novel adult-onset primary open-angle glaucoma (POAG) gene on 5q22.1. Hum Mol Genet 14: 725–733. 15677485 10.1093/hmg/ddi068

[15] Bartelt-Kirbach B, Wuepping M, Dodrimont-Lattke M, Kaufmann D (2009) Expression analysis of genes lying in the NF1 microdeletion interval points to four candidate modifiers for neurofibroma formation. Neurogenetics 10: 79–85. 18850118 10.1007/s10048-008-0154-0

[16] Stirnimann CU, Petsalaki E, Russell RB, Muller CW (2010) WD40 proteins propel cellular networks. Trends Biochem Sci 35: 565–574. 20451393 10.1016/j.tibs.2010.04.003

[17] Champion EA, Lane BH, Jackrel ME, Regan L, Baserga SJ (2008) A direct interaction between the Utp6 half-a-tetratricopeptide repeat domain and a specific peptide in Utp21 is essential for efficient pre-rRNA processing. Mol Cell Biol 28: 6547–6556. 18725399 10.1128/MCB.00906-08 PMC2573231

[18] Yang B, Wu YJ, Zhu M, Fan SB, Lin J, et al (2012) Identification of cross-linked peptides from complex samples. Nat Methods 9: 904–906. 22772728 10.1038/nmeth.2099

[19] Li T, Chen X, Garbutt KC, Zhou P, Zheng N (2006) Structure of DDB1 in complex with a paramyxovirus V protein: viral hijack of a propeller cluster in ubiquitin ligase. Cell 124: 105–117. 16413485 10.1016/j.cell.2005.10.033

[20] Voegtli WC, Madrona AY, Wilson DK (2003) The structure of Aip1p, a WD repeat protein that regulates Cofilin-mediated actin depolymerization. J Biol Chem 278: 34373–34379. 12807914 10.1074/jbc.M302773200

[21] Reubold TF, Wohlgemuth S, Eschenburg S (2011) Crystal structure of full-length Apaf-1: how the death signal is relayed in the mitochondrial pathway of apoptosis. Structure 19: 1074–1083. 21827944 10.1016/j.str.2011.05.013

[22] Charette JM, Baserga SJ (2010) The DEAD-box RNA helicase-like Utp25 is an SSU processome component. RNA 16: 2156–2169. 20884785 10.1261/rna.2359810 PMC2957055

[23] Tarassov K, Messier V, Landry CR, Radinovic S, Serna Molina MM, et al (2008) An in vivo map of the yeast protein interactome. Science 320: 1465–1470. 18467557 10.1126/science.1153878

[24] Van Duyne GD, Standaert RF, Karplus PA, Schreiber SL, Clardy J (1993) Atomic structures of the human immunophilin FKBP-12 complexes with FK506 and rapamycin. J Mol Biol 229: 105–124. 7678431 10.1006/jmbi.1993.1012

[25] Otwinowski Z, Minor W (1997) Processing of X-ray diffraction data collected in oscillation mode. Methods Enzymol 276: 307–326. 10.1016/S0076-6879(97)76066-X 27754618

[26] Vonrhein C, Blanc E, Roversi P, Bricogne G (2007) Automated structure solution with autoSHARP. Methods Mol Biol 364: 215–230. 17172768 10.1385/1-59745-266-1:215

[27] Sheldrick GM (2008) A short history of SHELX. Acta Crystallogr A 64: 112–122. 18156677 10.1107/S0108767307043930

[28] Emsley P, Cowtan K (2004) Coot: model-building tools for molecular graphics. Acta Crystallogr D Biol Crystallogr 60: 2126–2132. 15572765 10.1107/S0907444904019158

[29] Adams PD, Afonine PV, Bunkoczi G, Chen VB, Davis IW, et al (2010) PHENIX: a comprehensive Python-based system for macromolecular structure solution. Acta Crystallogr D Biol Crystallogr 66: 213–221. 20124702 10.1107/S0907444909052925 PMC2815670

[30] Murshudov GN, Vagin AA, Lebedev A, Wilson KS, Dodson EJ (1999) Efficient anisotropic refinement of macromolecular structures using FFT. Acta Crystallogr D Biol Crystallogr 55: 247–255. 10089417 10.1107/S090744499801405X

